# Widely Targeted Metabolomics Analyses Provide Insights into the Transformation of Active Ingredients During Drying and the Mechanisms of Color Change for Forest Ginseng (*Panax ginseng* C. A. Mey. cv. Sativi-nemoralis)

**DOI:** 10.3390/plants14030494

**Published:** 2025-02-06

**Authors:** Junjia Xing, Limin Yang, Lianxue Zhang, Jiahong Han, Enbo Cai

**Affiliations:** College of Chinese Medicinal Material, Jilin Agricultural University, 2888 Xincheng Street, Changchun 130118, China; hill_0307@163.com (J.X.);

**Keywords:** forest ginseng, widely targeted metabolomics, drying process, color

## Abstract

In this study, we investigated the mechanism of conversion of active components as well as the color change of forest ginseng (FG) during the drying process with the self-developed negative-pressure circulating airflow-assisted desiccator (PCAD) drying method, using a widely targeted metabolomics analytical method based on ultraperformance liquid chromatography–tandem mass spectrometry (UPLC-MS/MS). During the drying process, a total of 1862 metabolites were identified in FG, along with 748 differential abundant metabolites (DAMs). Further analysis of the types and metabolic pathways of the DAMs revealed that both primary and secondary metabolites changed by 50–70% moisture content (MC); secondary metabolites dominated with a 30–50% MC, and primary metabolites dominated with a 10–30% MC, which revealed the differences in the transformation of the active ingredients in the drying process. In addition, the results showed the browning characteristics during the drying process. MC-50 and MC-10 showed the smallest and largest color changes, as well as enzyme activities, compared to the other MCs, respectively. As drying proceeded, browning reactions were mainly related to lipid and nucleotide metabolism and phenylpropane and flavonoid biosynthesis. In conclusion, the present study provides theoretical support for the mechanisms of active ingredient transformation as well as the color change of FG during PCAD drying.

## 1. Introduction

Ginseng (*Panax ginseng* C. A. Mey) is a perennial herbaceous plant, which is a famous medicinal plant [1] and known as the king of herbs. Ginseng contains many active ingredients, including ginsenosides, sugars, organic acids, etc., which have antioxidant, anti-fatigue, anti-viral, anti-inflammatory and anti-tumor effects [2,3]. According to different growing environments and different cultivation methods, the Chinese Pharmacopoeia distinguishes two types of ginseng: garden ginseng (CG) and forest ginseng (FG). CG is artificially cultivated in gardens, whereas FG is grown naturally in mountain forests after artificial sowing, so that the active ingredients of FG are similar to those of the wild ginseng which has been grown for a long period of time, and according to the clinical experience of traditional Chinese medicine, it has a significantly higher medicinal value than GG.

Because FG is perishable and affected by the harvest season, consumers cannot purchase FG at any time, so dried FG is more popular among consumers. The quality of FG depends on many factors, among which the drying technique is particularly important [4]. Unreasonable drying processes can cause a gradual decrease in or even the disappearance of the content of active ingredients accumulated in the previous period [5,6]. The results of previous experiments have demonstrated that FG drying using the self-developed negative-pressure circulating airflow-assisted desiccator (PCAD) drying method can maximize the retention of the active ingredient content in FG [7]; therefore, it is necessary to elucidate the metabolite changes in FG during PCAD drying.

In addition, it has been found that the drying process is dominated by enzymatic reactions, in which water loss stress induces an increase in the activity of antioxidant enzymes, which in turn promotes the synthesis of a large number of secondary metabolites [8,9]. Common antioxidant enzymes include superoxide dismutase (SOD), catalase (CAT) and peroxidase (POD). Terpenoids are important secondary metabolites in FG, so it is equally essential to explore the activities of key rate-limiting enzymes (3-hydroxy-3-methylglutaryl coenzyme A reductase (HMGR), squalene synthase (SS) and dammarenediol synthase (DS)) of the pathways essential for terpenoid synthesis. It has been found that the enzyme activities in ginseng can be changed by external influences, which in turn have an impact on the quality [10]. These changes in quality can be reflected in the production and consumption of metabolites. In addition, enzymatic reactions often result in changes in the material color [11]. Therefore, the study of metabolite changes in FG could provide key information to understand the mechanism of color changes in FG during drying.

Metabolomics provides qualitative and quantitative analyses of all small molecules detected in a sample and helps to deepen the understanding of the mechanisms of chemical compositional transformation [12]. Widely targeted metabolomics has been utilized extensively to study compositional changes in different materials during drying due to its broad coverage, high throughput and sensitivity [13]; therefore, it is theoretically feasible to use widely targeted metabolomics to study the mechanisms of active ingredient transformations as well as color changes in the drying process of FG.

Therefore, the objectives of this study were (1) to reveal the metabolite transformations of FG during PCAD drying by using ultra-performance liquid chromatography–tandem mass spectrometry (UPLC-MS/MS) combined with a widely targeted metabolomics approach and (2) to investigate the relationship between metabolite alteration and browning during FG drying. Therefore, this study provides theoretical support for the transformation of active ingredients and the mechanism of the FG color change during PCAD drying.

## 2. Materials and Methods

### 2.1. Raw Materials

Fourteen-year-old ginseng root was harvested from the wild mountain ginseng base in Huanren Man Autonomous County, Benxi City, Liaoning Province, China. The roots were identified as *Panax ginseng* C. A. Mey. cv. Sativi-nemoralis by Prof. Yang Limin from the College of Chinese Medicinal Material at Jilin Agricultural University in Jilin, China.

### 2.2. Drying Method

**Figure 1:**
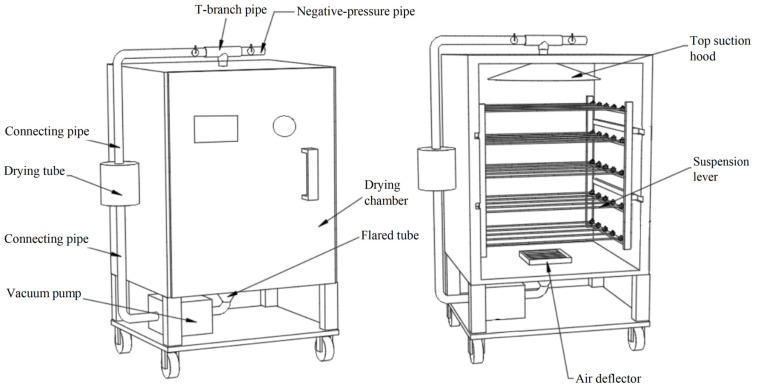
Drying system.

Negative-pressure circulating airflow-assisted desiccator drying: The negative-pressure circulating drying system used in the experiment was developed by the experimental group itself and has been patented [14]. As shown in Figure 1, the processed samples were evenly placed in the drying box, and the vacuum pump in the system was opened to remove the air in the drying chamber. At this time, the vacuum gauge showed 0.04 MPa, and the desiccant was placed in the drying tube. The circulation system (AP9925(N) vacuum pump, Tianjin Aotaisens Instrument Co., Ltd., Tianjin, China) was turned on, and the airflow speed in the drying box was detected as 4.7 m/s.

According to the initial moisture measurement of FG, the initial wet basis moisture content of FG was 70.15% ± 0.80. The FG was weighed several times in the drying process (the weighing process should be completed quickly, and the time should be controlled within 30 s), and samples were taken at a 50%, 30% and 10% moisture content, respectively, and quickly transferred to liquid nitrogen for freezing. Then, we further analyzed the indicators.

For the mass determination of the specific moisture content of FG, the calculation formula is as follows [15]:

(1) $$M_{X}=\left(1-\left(70\%-X\right)\right)\times M_{70\%}$$

*X* is the particular moisture content of FG, *M_X_* is the mass (g) of FG at a moisture content of *X* and *M*_70%_ is the mass (g) of FG at a moisture content of 70%.

### 2.3. Color

Color parameters were measured in 1 cm of the main root of the FG using a CR400 spectrophotometer (Konica Minolta Sensing, Tokyo, Japan), and three measurements were averaged. We calculated the total color change (Δ*E*) and browning index (*BI*) [11].

(2) $$\Delta E=\sqrt{{\left({\left(L_{0}^{*}-L_{1}^{*}\right)}^{2}+(a_{0}^{*}-a_{1}^{*}\right)}^{2}+{\left(b_{0}^{*}-b_{1}^{*}\right)}^{2})}$$

(3) $$BI=100\times \frac{\left(X-0.31\right)}{0.17}$$

(4) $$X=\frac{\left(a^{*}+{1.75L}^{*}\right)}{{6.645L}^{*}+a^{*}-{3.012b}^{*}}$$

In this formula, a^*^_0_, b^*^_0_ and L^*^_0_ are the redness/greenness, yellowness/blueness and brightness/darkness of the fresh sample, respectively, where a^*^_1_, b^*^_1_ and L^*^_1_ are the color values of dried FG.

### 2.4. Enzyme Activity Analyses

Ma et al.’s crude enzyme extraction method was referred to with minor modifications [16]. The 0.5 g sample was pulverized with liquid nitrogen, placed in a precooled mortar, added to 2.5 mL of phosphate-buffered saline (PBS) (pH 7.8), and ground into a homogenate. The homogenate was kept at a constant volume of 5 mL and centrifuged at 4000 r/min and 4 °C for 20 min, and the supernatant was collected at 4 °C for use.

#### 2.4.1. Antioxidant Enzyme Activity

The assessment of the enzymatic activity of SOD, POD and CAT in the FG were carried out using assay kits. These kits were purchased from Nanjing Jiancheng Bioengineering Institute (Nanjing, China). The enzyme activities were measured according to the manufacturer’s instructions. In brief, the POD activity was assayed based on the specific oxidation products that could be detected spectrophotometrically at a wavelength of 420 nm. SOD could scavenge O^2−^, the characteristic absorption of which could be detected at 550 nm according to the hydroxylamine method. The CAT activity was measured at 405 nm using the ammonium molybdate method.

#### 2.4.2. Activities of Terpenoid Biosynthesis-Related Enzymes

Enzyme-linked immunoassay (ELISA) kits (Andy Gene Co., Ltd., Beijing, China) were used to detect the activities of HMGR, SS and DS. According to the specific operation steps in the manual, a standard curve was drawn. The extracted samples of the crude enzyme solution of FG were accurately added. The OD values were read on the enzyme plate reader at 450 nm. The enzyme activity of each sample was calculated according to the standard curve.

### 2.5. Metabolomic Analysis [17]

#### 2.5.1. Metabolite Extraction/Dry Sample Extraction

Using vacuum freeze-drying technology, we placed the samples in a lyophilizer (Scientz-100F Ningbo scientz biotechnology Co., Ltd., Ningbo, China). The samples were ground to a powder form using a Retsch MM400 grinder (Verder Shanghai Instruments and Equipment Co., Ltd., Shanghai, China) (30 Hz, 1.5 min). Then, 50 mg of the powder was weighed using an electronic balance (MS105DM) and 1200 μL of a −20 °C precooled 70% methanolic aqueous internal standard extract was added to the sample. The mixture was vortexed for 30 s every 30 min for six repetitions. After vortexing, the sample was centrifuged (12,000 rpm, 3 min) and the supernatant was carefully aspirated. The sample was filtered through a 0.22 μm microporous membrane and transferred to an injection vial for UPLC-MS/MS analysis.

#### 2.5.2. UPLC-MS/MS

The sample extracts were analyzed using an UPLC-ESI-MS/MS system (UPLC, ExionLC™ AD, https://sciex.com.cn/, accessed on 19 September 2024) and tandem mass spectrometry system (https://sciex.com.cn/, accessed on 19 September 2024). The analytical conditions were as follows: UPLC: We used an Agilent SB-C18 column (1.8 µm, 2.1 mm × 100 mm); the mobile phase consisted of solvent A, pure water with 0.1% formic acid, and solvent B, acetonitrile with 0.1% formic acid. Sample measurements were performed with a gradient program that employed the starting conditions of 95% A and 5% B. Within 9 min, a linear gradient to 5% A and 95% B was programmed, and a composition of 5% A and 95% B was maintained for 1 min. Subsequently, a composition of 95% A and 5.0% B was adjusted for within 1.1 min and maintained for 2.9 min. The flow velocity was set as 0.35 mL per minute; the column oven was set to 40 °C; and the injection volume was 2 μL. The effluent was additionally connected to an ESI-triple-quadrupole linear ion trap (QTRAP)-MS.

The ESI source operation parameters were as follows: a source temperature of 500 °C; an ion spray voltage (IS) of 5500 V (positive-ion mode)/−4500 V (negative-ion mode); the ion source gas I (GSI), gas II (GSII) and curtain gas (CUR) were set at 50, 60 and 25 psi, respectively; and the collision-activated dissociation (CAD) was high. Triple-quadrupole (QQQ) scans were acquired during multiple reaction monitoring (MRM) experiments with the collision gas (nitrogen) set to medium. The DP (declustering potential) and CE (collision energy) for individual MRM transitions were set with further DP and CE optimizations. A specific set of MRM transitions were monitored for each period according to the metabolites eluted within this period.

#### 2.5.3. Qualitative and Quantitative Determination of Metabolites

For qualitative analysis, the self-built Metware database was used, and the retention time (RT), secondary mass spectrometry (all the fragment ions of the substance), molecular weight of the parent ion, characteristic fragment ion, DP and CE of the substance were matched with those of the substance in the database. The isotopic signals, duplicate signals containing K^+^, Na^+^ and NH_4_^+^ ions and the repeating signals of fragment ions from other larger molecular weight substances were excluded from the analysis.

The metabolites were quantified with the MRM mode using triple-quadrupole mass spectrometry. In the MRM mode, the precursor ions (parent ions) of the target substance were first filtered by the quadrupole to exclude interference from the ions of other substances. The precursor ions were fragmented to sub-ions in the collision chamber, which were further filtered through QQQ to select the characteristic target ion, thereby eliminating nontarget ion interference to make the quantification more accurate and reproducible. After obtaining the metabolite spectra of the different samples, the peak areas of the mass spectra of all the substances were integrated and corrected for the mass spectra of the same metabolite in different samples [18].

The identified metabolites were annotated using the KEGG database (https://www.kegg.jp/kegg/compound/, accessed on 8 October 2024), and the annotated metabolites were then mapped to the KEGG pathway database (https://www.kegg.jp/kegg/pathway.html, accessed on 8 October 2024). Then, the mapped pathways of the significantly regulated metabolites were fed into Metabolite Set Enrichment Analysis. The significance was determined by the *p*-values of the hypergeometric test.

### 2.6. Data Analysis

All analyses were carried out in triplicate, with a sample size of 20 roots per group. All result values are given as the average value with the standard deviation obtained using SPSS 23.0 software (SPSS Inc., Chicago, IL, USA). The verification of significant differences was performed using Duncan’s multiple-range test (*p* < 0.05) after an ANOVA. Each value is a mean ± the S.D. Graphing was conducted using Origin Lab software (https://www.originlab.com, accessed on 25 September 2024, OriginLab Corp., Northampton, MA, USA). An unsupervised principal component analysis (PCA) was performed using the statistics function prcomp within R (https://www.r-project.org accessed on 25 September 2024). The data were unit variance-scaled before performing the unsupervised PCA. The HCA (hierarchical cluster analysis) results of the samples and metabolites were presented as heatmaps with dendrograms. The HCA was carried out by the R package ComplexHeatmap. For HCA, the normalized signal intensities of the metabolites (unit variance scaling) were visualized as a color spectrum. For two-group analysis, differential metabolites were determined using variable importance in projection (VIP) scores (VIP ≥ 1) and absolute values of Log_2_FC (|Log_2_FC| ≥ 1.0). The VIP values were extracted from the orthogonal partial least squares-discriminant analysis (OPLS-DA) results (which also contained score plots), and permutation plots were generated using the R package 1.0.1 MetaboAnalystR. The data were in log transform (log_2_) form and mean-centered before OPLS-DA. A permutation test (200 permutations) was performed to avoid overfitting.

## 3. Results

### 3.1. Color Attributes

**Figure 2:**
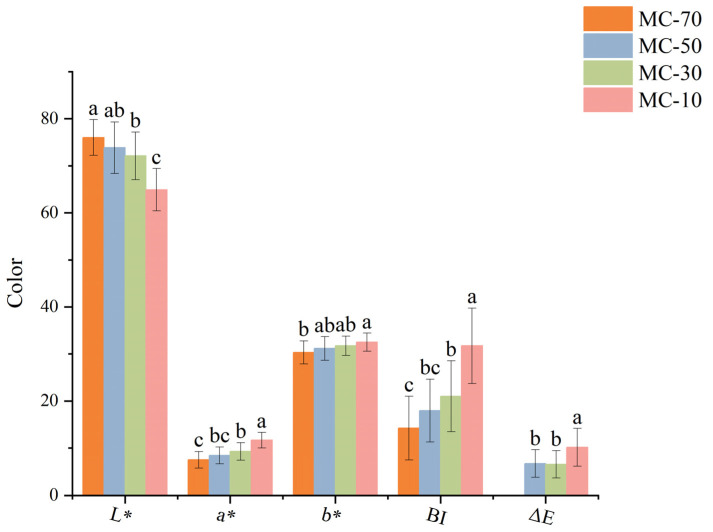
Color parameters of forest ginseng. a*, b* and L* are the redness/greenness, yellowness/blueness and brightness/darkness of the samples with different water contents. Δ*E*: color change; *BI*: browning index. Different letters represent significant differences between the groups (*p* < 0.05). Identical letters indicate that there was no significant difference between the groups (*p* > 0.05).

The process of the color change of FG at four different moisture contents during PCAD drying was subjected to study and comparisons. As the water content decreased, the FG as a whole exhibited a notable decline in L* and an increase in a* and b* (Figure 2), thereby indicating that the drying process exerted a considerable influence on the color of FG. In this case, L* decreased significantly and a^*^ increased significantly at MC-10 compared to those of the other moisture contents, indicating a darkening of the FG’s color when the moisture content decreased from 30% to 10%. The *BI* and Δ*E* values exhibited notable increases of 51.10–122.69% and 51.17–54.91%, respectively, following the drying process when compared to those of the other moisture contents. The data indicated that the color of FG underwent a significant change at MC-10. Furthermore, the data demonstrated that neither L*, a* and b* nor *BI* or Δ*E* exhibited a notable color change at MC-50 vs. MC-30, suggesting that the color change was not significant when the moisture content was reduced from 50% to 30%.

### 3.2. Activities of Enzymes

**Figure 3:**
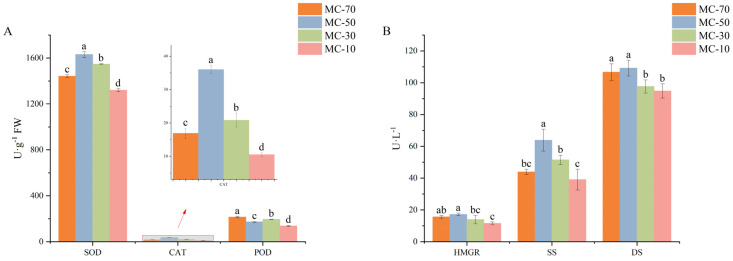
Changes in enzyme activity during drying. (**A**) Antioxidant enzyme activity; (**B**) activities of terpenoid biosynthesis-related enzymes. Different letters represent significant differences between groups (*p* < 0.05). Identical letters indicate that there was no significant difference between groups (*p* > 0.05).

The initial levels of the six enzyme activities were high, but they subsequently exhibited fluctuations in response to the drying process before showing a notable decline (Figure 3A,B). With the exception of POD, five enzyme activities demonstrated a pattern of an initial increase followed by a decline. All five exhibited the highest enzyme activity at MC-50. This suggests that FG was subjected to a considerable external stress stimulus during the pre-drying phase, which subsequently augmented the enzyme activities in FG. This may be a pivotal point of investigation with regard to the drying process of the PCAD. In comparison to the other water contents, SOD, CAT and SS exhibited notable increases of 5.39–23.43%, 72.96–243.75% and 24.06–63.45%, respectively, at MC-50. This suggests that the aforementioned enzymes demonstrated a heightened sensitivity to the water content in FG. Subsequently, the enzyme activities of SOD, CAT, HRGM, SS and DS exhibited a gradual decline with a reduction in the water content, reaching a nadir at MC-10%. In contrast, the POD enzyme demonstrated an ‘S’-shaped trend, initially decreasing, then increasing and subsequently decreasing once more. This also resulted in the lowest enzyme activity after drying (MC-10%), indicating that the reduction in the water content had a pronounced impact on the enzyme activity.

### 3.3. Metabolome Analysis of Dried FG

#### 3.3.1. Metabolite Overview of Dried FG

**Figure 4:**
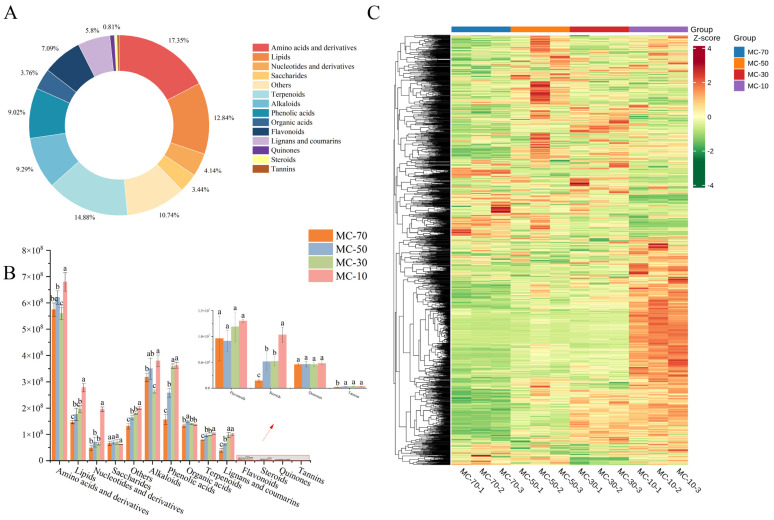
Metabolomics analysis of FG. (**A**) Metabolite classification of FG; (**B**) comparison of metabolite classes in four samples; (**C**) cluster analysis of all metabolites in sample. Different letters represent significant differences between groups (*p* < 0.05). Identical letters indicate that there was no significant difference between groups (*p* > 0.05).

A total of 1862 metabolites were identified in FG samples treated with different water contents (Table S1). The most diverse were amino acids and derivatives (323), terpenoids (277), lipids (239), alkaloids (173), phenolic acids (168), flavonoids (132) and lignans and coumarins (108) (Figure 4A); there were 77 nucleotides and derivatives, 70 organic acids, 64 saccharides, 15 quinones, 9 steroids, 7 tannins and 200 other compounds.

The metabolites were quantified using the MRM mode, and their abundance was calculated using the signal strength of the mass spectra. The metabolites were quantitatively compared by their category (Figure 4B). The highest abundance of metabolites was observed at MC-10 in comparison to that of the other water contents. Furthermore, the content of MC-10 dried samples was higher than that of the other water contents, with the exception of saccharides. In this case, sterols, nucleotides and derivatives, lipids, amino acids and derivatives and terpenoids were significantly higher by 96.80–605.13%, 177.36–305.95% and 42.38–90.29%, respectively, and 9.38–21.20% and 7.22–32.76%, respectively. The organic acid content was observed to increase by 6.04–10.70% at MC-50 in comparison to that of the other moisture contents. The highest concentrations of phenolic acids, lignin and coumarin were observed at MC-30 and MC-10, although the differences were not statistically significant. The lowest concentrations of amino acids and alkaloids were observed at MC-30, with reductions of 2.59–21.20% and 20.71–44.55%, respectively. These findings indicate that variations in the water content influence the composition and concentration of species within FG.

The HCA thermograms (Figure 4C) demonstrate a clear hierarchical cluster analysis among the four groups. It is obvious that the metabolites in the varied water contents exhibited considerable variances. This suggests that the types and contents of metabolites in the four groups underwent a notable transformation throughout the drying process. The overall analysis revealed a notable increase in the metabolite content during the drying process, suggesting that the FG stress response to the drying process was intensified with a reduction in the water content.

#### 3.3.2. PCA and OPLS-DA

**Figure 5:**
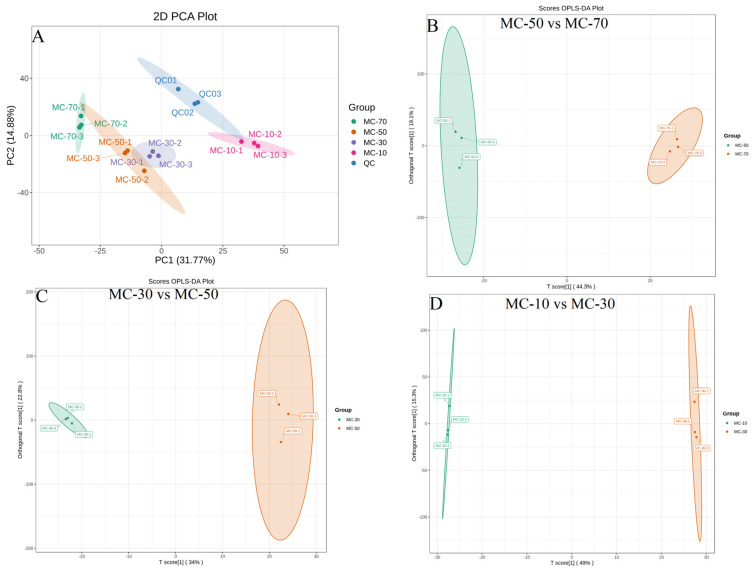
Results of the PCA and OPLS-DA. (**A**) Plot of the PCA model of different FG samples, including QC samples. (**B**–**D**) The OPLS-DA score plots of the differential metabolites in the three pairwise comparisons.

The chemical composition of FG was analyzed using a widely targeted metabolomic approach based on UPLC-MS/MS, and the extracted ion chromatograms of the quality control (QC) samples and internal standard are presented in Figures S1 and S2. PC1 and PC2 collectively explained 46.65% of the total variance (Figure 5A). An unsupervised PCA is a method of the statistical analysis of multidimensional data commonly used to investigate how a small number of principal components can reveal the internal structure among multiple variables. PCA effectively differentiated between the dry (MC-10) and fresh (MC-70, MC-50, MC-30) samples, indicating that drying significantly altered the metabolites in the FG. The distribution of metabolites was more clustered in the QC samples as well as in each of the treatment groups, indicating that the metabolites were more concentrated within the groups. There was an overlap between the MC-50 and MC-30 samples, suggesting that the metabolite changes in FG were similar at higher water contents. The OPLS-DA model demonstrated a clear separation of the MC-70, MC-50, MC-30 and MC-10 groups (Figure 5B,C). The MC-50 and MC-70 groups exhibited high predictability (Q^2^) and a strong goodness-of-fit (R^2^X, R^2^Y) (Q^2^ = 0.888, R^2^X = 0.634, R^2^Y = 0.999, Figure S3A), as did the MC-30 and MC-50 groups (Q^2^ = 0.835, R^2^X = 0.569, R^2^Y = 0.999, Figure S3B) and the MC-10 and MC-30 groups (Q^2^ = 0.922, R^2^X = 0.643, R^2^Y = 1, Figure S3C). These findings suggest that the OPLS-DA model was stable and highly reliable for this dataset, thereby providing support for the validity of the model in the further screening of different metabolites.

#### 3.3.3. Differentially Abundant Metabolites (DAMs)

**Figure 6:**
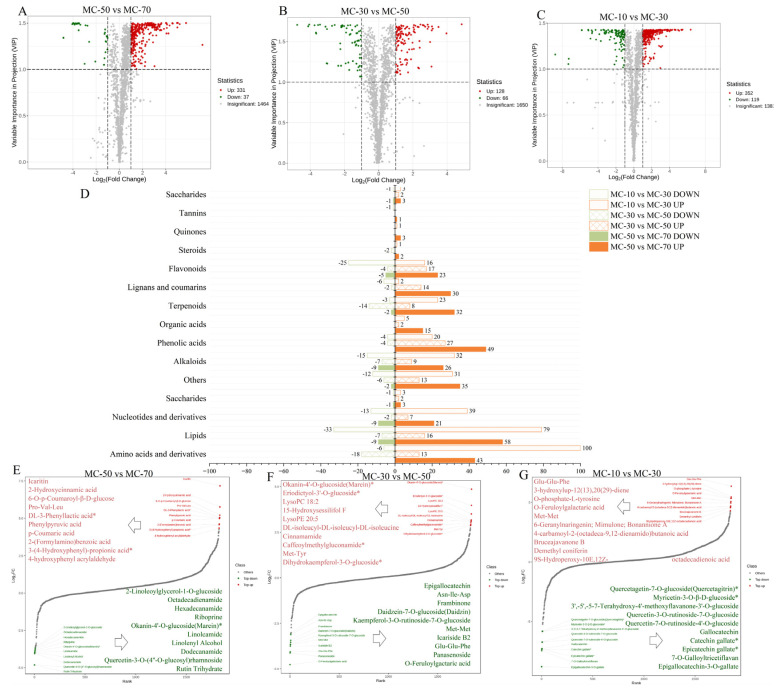
DAM analysis of the FG. (**A**–**C**) Volcano plots showing the expression levels of the DAMs in the three pairwise comparisons. Green dots represent down-regulated differentially expressed metabolites; red spots represent up-regulated differentially expressed metabolites; and gray spots represent non-differentially expressed metabolites. (**D**) The number of differentially expressed metabolites of three pairwise comparisons in FG. Orange columns represent up-regulated differentially expressed metabolites; green columns represent down-regulated differentially expressed metabolites. The numbers located on the side of the columns represent the number of differentially expressed metabolites of each pairwise comparison of FG. (**E**–**G**) * The bar chart of the top 10 substances corresponding to the difference change of the differentiated metabolites in the group.

In order to gain further insight into the metabolic differences observed in the pairwise comparisons, all 1862 metabolites were subjected to screening in order to identify those that exhibited DAMs. Metabolites with a VIP value of ≥1 and an FC of ≥2 (or an FC of ≤0.5) were selected as DAMs. The volcano plots (Figure 6A–C) demonstrated that there were 368 DAMs (331 up-regulated and 37 down-regulated) in the comparison between the MC-50 and MC-70 groups, 194 DAMs (128 up-regulated and 66 down-regulated) in the comparison between the MC-30 and MC-50 groups and 471 DAMs (352 up-regulated and 119 down-regulated) in the comparison between the MC-10 vs. MC-30 groups. The number of up-regulated metabolites increased relative to the number of down-regulated metabolites, suggesting that the drying process had a significant effect on the physiological metabolic activity of FG metabolites. The number of significantly different metabolites was higher in the MC-50 vs. MC-70 group and in the MC-10 vs. MC-30 group than in the MC-30 vs. MC-50 group, suggesting that the significant effect of the drying process on the physiological metabolic activity of FG metabolites in the process of decreasing the water content from 70% to 50% and from 30% to 10% was higher than that of the decrease from 50% to 30% water content.

The DAMs produced during FG drying were subjected to further classification and comparison. The DAMs were classified into 14 categories (Figure 6D), with amino acids and derivatives, nucleotides and derivatives, lipids and organic acids representing the major metabolites of the plant and phenolic acids, flavonoids, lignans and coumarins, alkaloids and terpenoids representing the minor metabolites. The DAMs were predominantly concentrated in amino acids and derivatives, lipids, phenolic acids and alkaloids. The results demonstrated that the types and quantities of the DAMs of FG with different water contents were markedly distinct in response to the water loss treatment, indicating that FG with different water contents experienced disparate external stress conditions and exhibited differential physiological responses. During the drying process, a significant up-regulation of primary and secondary metabolites was observed when the water content was reduced from 70 to 50%. However, sterols, tannins, quinones and saccharides exhibited minimal changes. Lipids, phenolic acids and amino acids and derivatives were the most significantly affected metabolites. When the water content was reduced from 50 to 30%, the metabolites produced were phenolic acids, flavonoids and lipids. This resulted in the secondary metabolites being up-regulated at this stage. The up- and down-regulation of secondary metabolites was more pronounced than that of primary metabolites. The analysis of FG during a decrease from a 30 to a 10% water content revealed that the up-regulation of DAMs was primarily observed in amino acids and derivatives, lipids and nucleotides and derivatives (primary metabolites) and terpenoids, alkaloids, flavonoids and phenolic acids (secondary metabolites). This suggests that the process of drying had a significant impact on the production of metabolites.

In order to show the overall metabolic differences more clearly and visually, the dynamic distribution of metabolite content differences was plotted (Figure 6E–G), and the top 10 metabolites before up-regulation and down-regulation were labeled, and it was found that amino acids and derivatives and phenolic acids experienced significant up-regulation and nucleotides and derivatives, flavonoids, alkaloids and lipids significant down-regulation in the MC-50 vs. MC-70 group. In the MC-30 vs. MC-50 group, flavonoids, alkaloids, terpenoids and lipids experienced significant up-regulation and amino acids and derivatives and phenolic acids significant down-regulation. The significant up-regulation of amino acids and derivatives, phenolic acids, terpenoids and lipids was found in the MC-10 vs. MC-30 group; flavonoids were significantly down-regulated. These results indicated that the chemical components of FG were transformed during drying, mainly in the transformations of flavonoids, phenolic acids, amino acids and derivatives, lipids, alkaloids, terpenoids and nucleotides and derivatives, and these components were transformed by different mechanisms during different drying processes.

**Figure 7:**
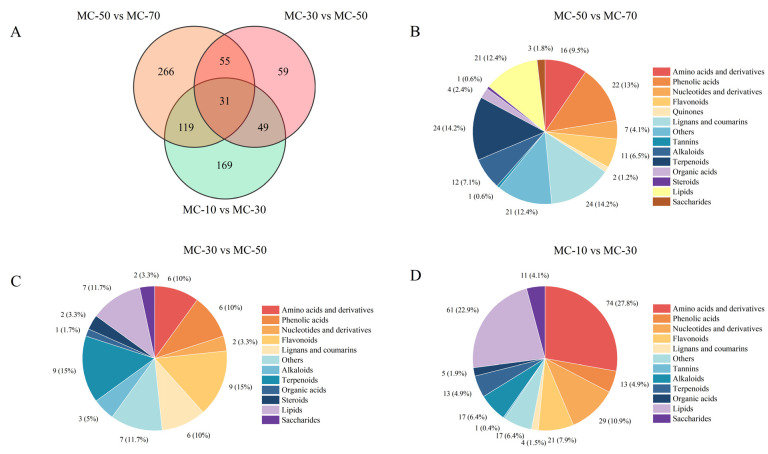
Differences in the number of DAMs between groups and the classification of unique DAMs. (**A**) Venn diagram of the differences in the number of DAMs between the three comparison groups; (**B**–**D**) the classification of the unique DAMs.

A Venn diagram was employed to identify the shared and unique metabolites of FG at varying stages of drying. A total of 748 DAMs were obtained from the four treatment groups of MC-70, MC-50, MC-30 and MC-10 (Figure 7A). Of these, 169 were identified as unique DAMs in the comparison between MC-50 and MC-70, 59 were unique DAMs in the comparison between MC-30 and MC-50 and 266 were unique DAMs in the comparison between MC-10 and MC-30. The high percentage of lignin and coumarin (n = 24), terpenoids (n = 24), phenolic acids (n = 22), lipids (n = 21) and amino acids and derivatives (n = 16) among the DAMs in the MC-50 vs. MC-70 group (Figure 7B) indicates that the DAMs at a decreasing water content from 70 to 50% mainly affected the above five types of compounds. The MC-30 vs. MC-50 DAMs included flavonoids (n = 9), terpenoids (n = 9), lipids (n = 7), phenolic acids (n = 6), lignans and coumarins (n = 6) and amino acids and derivatives (n = 6) (Figure 7C); it was observed that the DAMs at a decreasing water content from 50 to 30% primarily influenced the aforementioned six types of compounds. The MC-10 vs. MC-30 DAMs included amino acids and derivatives (n = 74), lipids (n = 61) and nucleotides and derivatives (n = 29) (Figure 7D), which indicated that DAMs at a decreasing water content from 30 to 10% mainly affected the above three types of compounds (primary metabolites). This result further confirmed that the reductions in the water content from 70 to 50% and from 30 to 10% were of particular significance for the conversion of metabolites during FG drying.

Ginsenosides, among terpenoids, are important active components of FG, so it was important to study the metabolic changes of this group during the drying process. The results showed (Table S2) that a total of forty-two ginsenosides were detected in the four groups, of which more than half were highest at MC-10, followed by MC-30 and MC-50, and lowest at MC-70, and only three ginsenosides were highest at MC-70, suggesting that drying was beneficial for the retention of the ginsenoside content. Interestingly, although the content of most ginsenosides increased after drying, different trends were observed. Some of the ginsenoside (e.g., ginsenoside Rh_4_, ginsenoside Rh_8_, pseudoginsenoside RT_5_ and ginsenoside Rf_1_) contents showed a gradual increase with a decreasing water content; some of the ginsenosides (e.g., finsenoside Rg_7_, ginsenoside Re_5_, ginsenoside Rh_15_, malonylginsenoside Rb_1_ and pseudoginsenoside Rt_4_) increased at MC-50, decreased at MC-30, and finally increased again at MC-10; and some of the ginsenosides (e.g., ginsenoside Rd_2_, ginsenoside Ro, ginsenoside Rg_1_, ginsenoside Rg_3_ and ginsenoside Rh_1_), in contrast to the former, showed a decreasing and then increasing trend. These phenomena further indicated that the drying process had a great effect on the ginsenosides in FG.

#### 3.3.4. Metabolic Pathway Analysis

**Figure 8:**
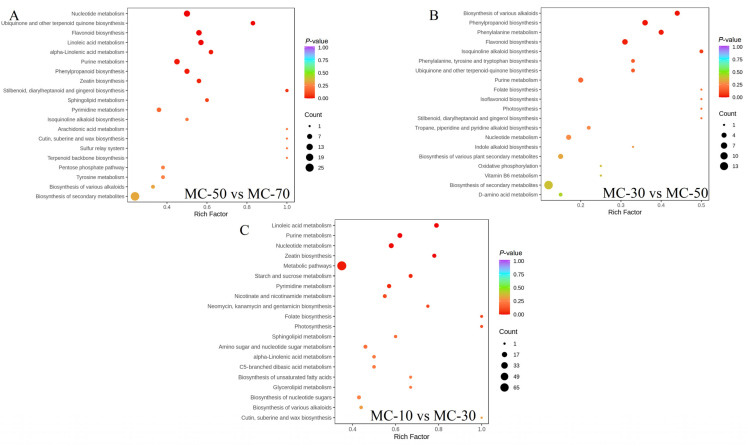
Metabolomic enrichment pathway analysis. (**A**) MC-50 vs. MC-70, (**B**) MC-30 vs. MC-50 and (**C**) MC-10 vs. MC-30.

To gain further insight into the biological mechanisms underlying these DAMs in FG, a KEGG pathway enrichment analysis was conducted. The results demonstrated that a total of 748 DAMs were identified, with the KEGG enrichment analysis encompassing a total of 79 pathways. Of the DAMs, 368 of the MC-50 vs. MC-70 group were found to be primarily involved in 46 KEGG metabolic pathways (Figure S4), 149 of the MC-30 vs. MC-50 group were found to be primarily involved in 38 KEGG metabolic pathways (Figure S5) and 471 of the MC-10 vs. MC-30 group were found to be primarily involved in 73 KEGG metabolic pathways (Figure S6). Following statistical analysis, it was determined that 18 metabolic pathways were involved in the three groups, with the majority of DAMs assigned to metabolic pathways and the biosynthesis of secondary metabolites. We constructed bubble plots showing the top 20 KEGG-enriched metabolic pathways (Figure 8A–C). Twenty metabolic pathways were identified with a *p* < 0.05 (nine, MC-50 vs. MC-70; four, MC-30 vs. MC-50; and seven, MC-10 vs. MC-30). These pathways focused on secondary metabolism: phenylpropanoid biosynthesis, phenylalanine metabolism, metabolic pathways, ubiquinone and other terpenoid quinone biosynthesis and the biosynthesis of various alkaloids, as well as primary metabolisms such as starch and sucrose metabolism, alpha-linolenic acid metabolism, nucleotide metabolism, pyrimidine metabolism, purine metabolism and linoleic acid metabolism. In addition, they were also involved in zeatin biosynthesis and stilbenoid, diarylheptanoid and gingerol biosynthesis. Overall, the above 20 pathways were found to be the most important metabolite pathways on the basis of their *p*-value and enrichment number. These enriched metabolic pathway-related metabolites reflect a possible mechanism by which the drying process affects FG metabolites, and therefore, drying regulates the FG quality by modulating the relevant significant DAMs of the above pathways.

## 4. Discussion

The color and active substances of FG undergo alteration during the drying process. In this study, a total of 1862 metabolites were obtained from the drying process of FG using the UPLC-MS/MS metabolomics method. These metabolites were subsequently identified and classified into 14 different categories. Therefore, the metabolite composition of FG is complex and diverse. Pairwise comparisons of samples subjected to different drying processes revealed that the drying process exerted disparate effects on the metabolites of FG, which in turn influenced the observed changes in color. Nevertheless, further analysis of the data demonstrated that the quantities and concentrations of numerous metabolites exhibited notable discrepancies between each group, indicating that the drying process exerted disparate influences on the formation of primary and secondary metabolites.

Browning is considered to be a combination of free radical oxidative species damage and enzymatic oxidation, among other things [19]. When the plant body is affected by unfavorable factors, the organism will produce a large number of reactive oxygen radicals, causing damage to the organism, and in order to slow down this damage, SOD, CAT and POD in the plant body will act in combination to maintain cellular homeostasis by converting the toxic reactive oxygen radicals into non-toxic water and oxygen [20,21]. However, POD is a peroxidase, and some researchers have suggested that the browning reaction may be partially attributed to the action of POD on polyphenols [22] and that the activity of POD exhibits an ‘S’ trend of decreasing, then increasing, and then decreasing at different water contents. It has been shown that the high activity of SOD and CAT largely lead to less susceptible organisms browning [11]. The results showed that the activities of SOD and CAT were highest at MC-50, and then the enzyme activities gradually decreased with a decreasing water content. The color results showed that FG exhibited the smallest and the largest chromaticity changes at MC-50 and MC-10, respectively, indicating that the magnitude of the enzyme activity changes matched to a certain extent with the FG browning and chromaticity indices, suggesting that it involved complex metabolic regulation.

Currently, many studies are demonstrating the correlation between enzymatic browning and the overall physiological metabolism of plants. Enzymatic browning was found to be largely associated with lipid metabolism [23]. Lipids play many important physiological roles, such as producing membranes, storing energy and forming signaling molecules [24]. The metabolomic results showed that lipids were the major metabolic components of FG, which is consistent with the results found by Zhang et al. [25], where free fatty acids (FAAs), glycerol ester (GL), lysophosphatidylcholine (LPC) and lysophosphatidylethanolamine (LPE) were the major lipid types. Desiccation causes excessive free radical production, leading to increased membrane lipid peroxidation and increased cell membrane permeability [26], which indirectly contribute to enzymatic browning [27,28], whereas higher relative concentrations of unsaturated fatty acids (e.g., linoleic and linolenic acids) help to maintain the structural integrity and stability of the cellular membranes that impede browning reactions. Thus, the lipid metabolisms of α-linolenic acid metabolism and linoleic acid metabolism contribute to the slowing down of browning. The contents of linoleic and linolenic acids increased with a decreasing water content in this study, further demonstrating the effect of browning due to drying on lipid metabolism. In addition, most of the GLs and FFAs increased with a decreasing water content, while most of the LPCs and LPEs decreased with a decreasing water content. These phenomena are contrary to the results of Zeng et al. [29] and may be related to the mode of drying. Zeng et al. found that heat-induced processes may promote the formation of LPC and LPE; therefore, it can be inferred that non-thermal drying processes may inhibit the formation of LPC and LPE, as well as promote the release of GL and FFAs.

Nucleotide metabolism plays a direct role in maintaining cellular homeostasis. Purines and pyrimidines are required for primary and secondary metabolism, which are basic cellular biochemical processes necessary for cell growth [30]. The adenosine/adenine pathway is thought to be effective in maintaining high levels of adenosine triphosphate (ATP) during plant damage, and altering purine levels can make plants more resistant to stress and promote sensitivity to stress [31]. Thus, when plants are damaged, the purine pathway usually becomes active. The present study is in agreement with the above findings as the levels of most of the metabolites involved in the purine pathway increased with a decreasing water content during drying, with significantly higher levels of guanosine, adenosine and adenine, suggesting that nucleotide metabolism also has an effect on browning. In addition, the accumulation of cytidine, which is thought to potentially prevent browning, was highly enriched in the pyrimidine metabolic pathway depending on the relative content of the metabolite.

High levels of the secondary metabolites of phenylpropane biosynthesis and flavonoid biosynthesis contribute to resistance to browning. It has been stated that phenylpropane biosynthesis is the main induced metabolism for browning inhibition, while flavonoid biosynthesis is an important endogenous pathway for browning resistance [32]. The phenylpropane biosynthesis pathway is located upstream of the flavonoid biosynthesis pathway and can lead to the biosynthesis of coumarins, flavonoids, isoflavones and flavanols [33]. The phenylpropane pathway may be enhanced due to desiccation-induced biostress [34], which in turn promotes the higher biosynthesis of flavonoids, thus providing protection to plants [35,36]. Flavonoids are the major secondary metabolites derived from phenylpropanes [37], and their accumulation protects plants from oxidative damage by scavenging free radicals [38]. The stimulation of the phenylpropane pathway and consequently the production of specific phenolic acids (including chlorogenic acid, 5-O-caffeoylmangiferic acid and p-coumaroylquinic acid) and flavonoids (including hesperidin and gallocatechin) has a significant effect on the prevention of browning [11], and the metabolomic results showed that the content of all the above-mentioned compounds (except for gallocatechin) increased with a decrease in the water content, which implied that the stress response generated by drying was the reason influencing the FG color change. Polyphenols are the most abundant secondary metabolites in plants, which not only play a defensive role by scavenging oxygen free radicals but also act as structural components to strengthen cell walls and reinforce mechanical barriers, thus delaying the onset of browning [39,40,41,42].

Ginsenosides belong to the triterpenoid saponins of the terpenoids and are produced via the mevalonate pathway catalyzed by terpenoid biosynthesis-related enzymes. The terpenoid biosynthesis-related enzyme activity showed a trend of increasing and then decreasing with a decreasing water content. This change may have been related to the drought stress response, which in turn promotes the production of the precursors needed for ginsenosides. The content of ginsenosides changed as the water content decreased. It has been shown that ginsenosides such as Rb_1_ may be converted to different ginsenosides such as F_2_, Rd, Rh_2_, etc., by enzymes [43], and a similar phenomenon was observed in this study. Therefore, it is hypothesized that the changes in the ginsenoside content at different drying stages may be related to the above-mentioned reasons, and these changes mainly occurred at the MC-50 and MC-30 stages, suggesting that these stage are the main stages affecting ginsenosides and also that these are important stages for the changes in other metabolites. In addition, terpenoids regulate the oxidative state of plants by reacting directly with intracellular or extracellular oxidants and indirectly by altering reactive oxygen species (ROS) signaling, thereby mitigating the effects of oxidative stress [44]. Therefore, the accumulation of terpenoids in FG contributes to stress tolerance and ameliorates browning.

There were still some limitations in this study. The other main active ingredient of FG is the volatile component. In addition to color, PCAD drying can better retain the odor of FG compared to conventional hot air drying. However, UPLC-MS/MS analysis is of limited value in detecting volatile components. In future studies, changes in the FG flavor during PCAD drying should be analyzed, with a focus on volatile components.

## 5. Conclusions

In this study, the mechanism of active ingredient transformation as well as the color change of FG during PCAD drying was investigated for the first time using a widely targeted metabolomics approach based on UPLC-MS/MS. The results showed that a total of 1862 metabolites were detected in four different water content samples during FG drying, and the major metabolites included amino acids and their derivatives, terpenoids, lipids, alkaloids, phenolic acids, flavonoids and lignans and coumarins, and 748 DAMs were screened and classified into 14 categories, and the pairwise comparisons showed that the differences between those metabolites detected at a water content of 50–70% and those detected at a 10–30% water content were significantly higher than those detected at a water content of 30–50%, and the further joint analysis of 20 significant KEGG pathways revealed that both primary and secondary metabolites changed at a water content of 50–70%, changes in the secondary metabolites predominated at a water content of 30–50% and changes in the primary metabolites predominated at a water content of 10–30%, which determined the material basis of the FG. The color change of FG is related to the enzyme activity associated with browning during drying. MC-50 and MC-10 showed the smallest and largest color changes as well as enzyme activities, respectively, compared to those of the other moisture contents. As drying proceeded, the browning reactions were mainly related to lipid metabolism, nucleotide metabolism, phenylpropane biosynthesis and flavonoid biosynthesis, suggesting that FG underwent different metabolic reactions during drying. In conclusion, this study provides theoretical support for the mechanisms of active ingredient transformation as well as the color change of FG during PCAD drying.

## Data Availability

The data that support the findings of this study are available from the corresponding author upon reasonable request. The data are not publicly available due to privacy.

## Supplementary Materials

The following supporting information can be downloaded at: https://www.mdpi.com/article/10.3390/plants14030494/s1.
