# Supplementary material for: Widely Targeted Metabolomics Analyses Provide Insights into the Transformation of Active Ingredients During Drying and the Mechanisms of Color Change for Forest Ginseng (Panax ginseng C. A. Mey. cv. Sativi-nemoralis)
Source: Plants (Basel). 2025 Feb 6;14(3):494. doi: 10.3390/plants14030494 (PMC11820357; doi:10.3390/plants14030494)
Supplement: Supplementary file 1 [file plants-14-00494-s001.zip › plants-3385705-supplementary/Figures Supplementary.pptx]

## Slide 1
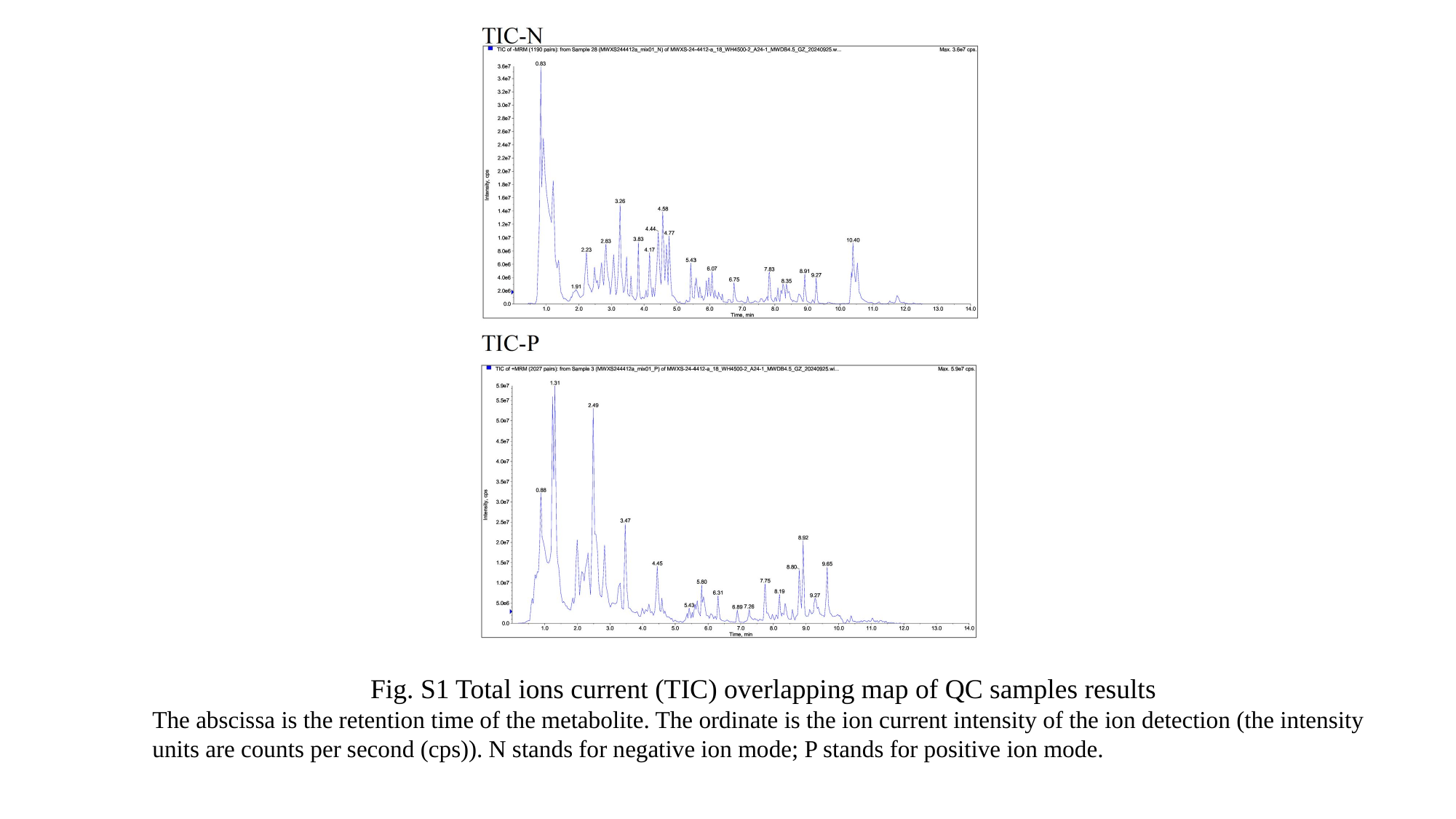

Fig. S1 Total ions current (TIC) overlapping map of QC samples results
The abscissa is the retention time of the metabolite. The ordinate is the ion current intensity of the ion detection (the intensity units are counts per second (cps)). N stands for negative ion mode; P stands for positive ion mode.

## Slide 2
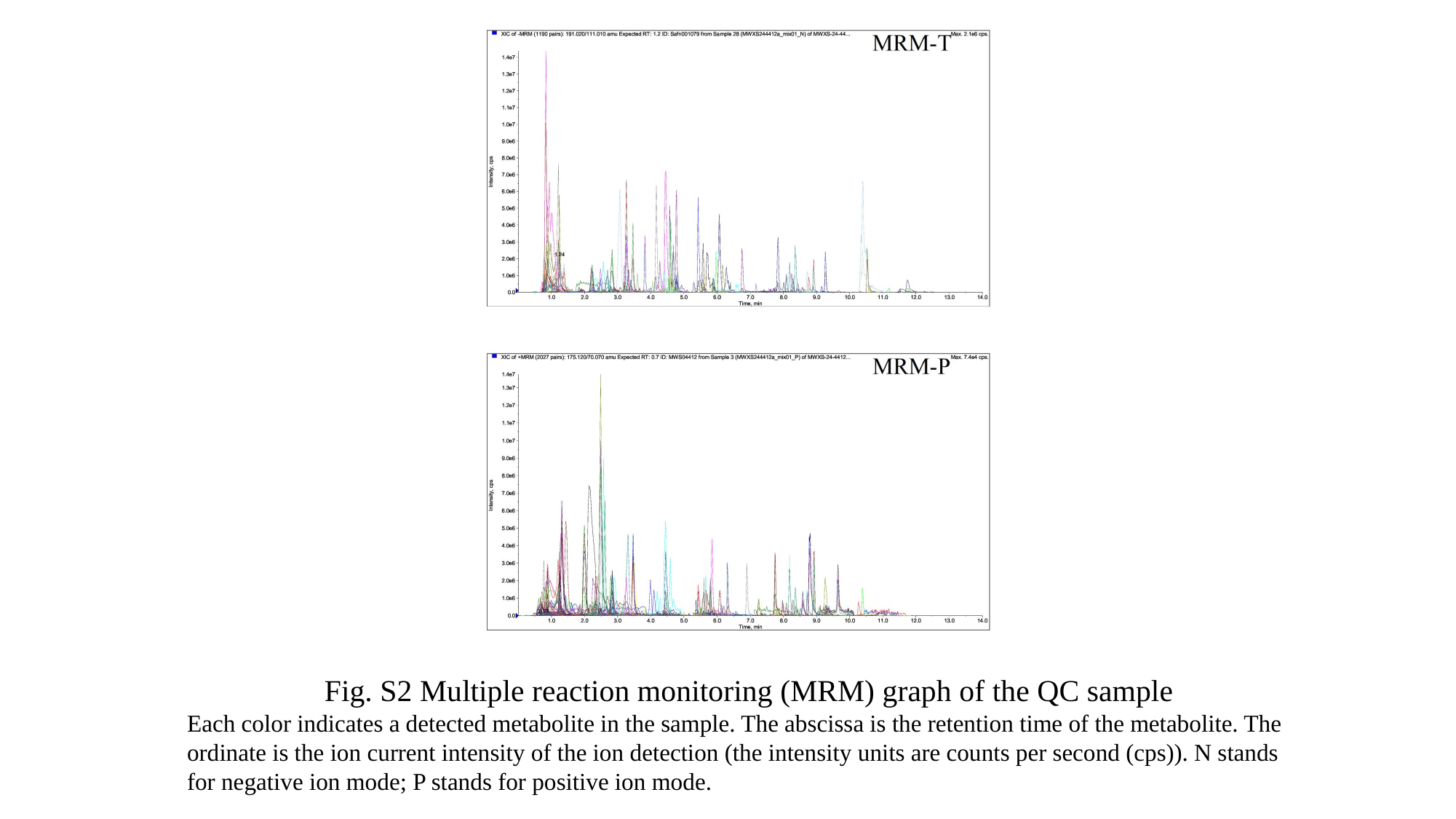

Fig. S2 Multiple reaction monitoring (MRM) graph of the QC sample
Each color indicates a detected metabolite in the sample. The abscissa is the retention time of the metabolite. The ordinate is the ion current intensity of the ion detection (the intensity units are counts per second (cps)). N stands for negative ion mode; P stands for positive ion mode.

## Slide 3
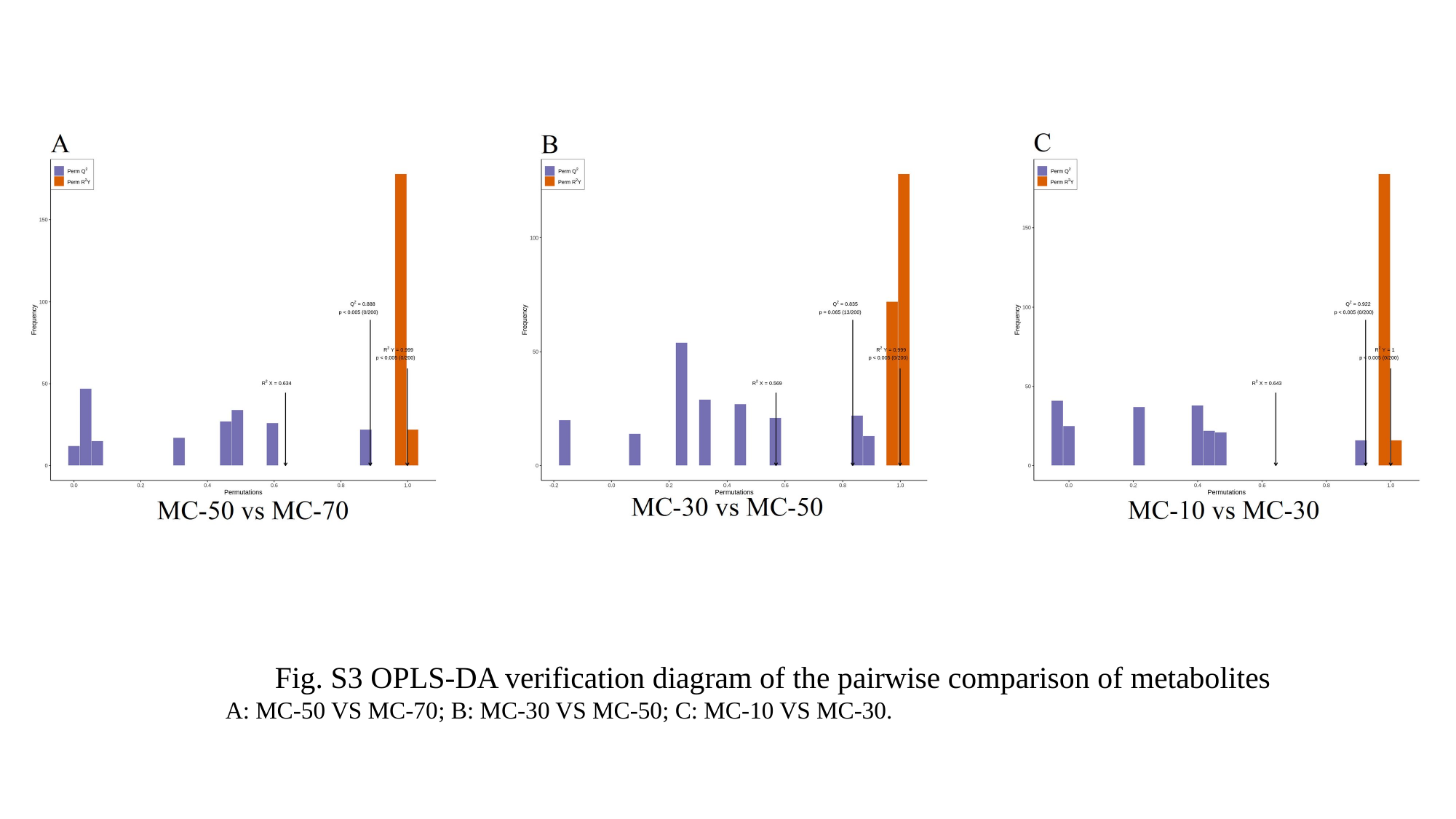

Fig. S3 OPLS-DA verification diagram of the pairwise comparison of metabolites
A: MC-50 VS MC-70; B: MC-30 VS MC-50; C: MC-10 VS MC-30.

## Slide 4
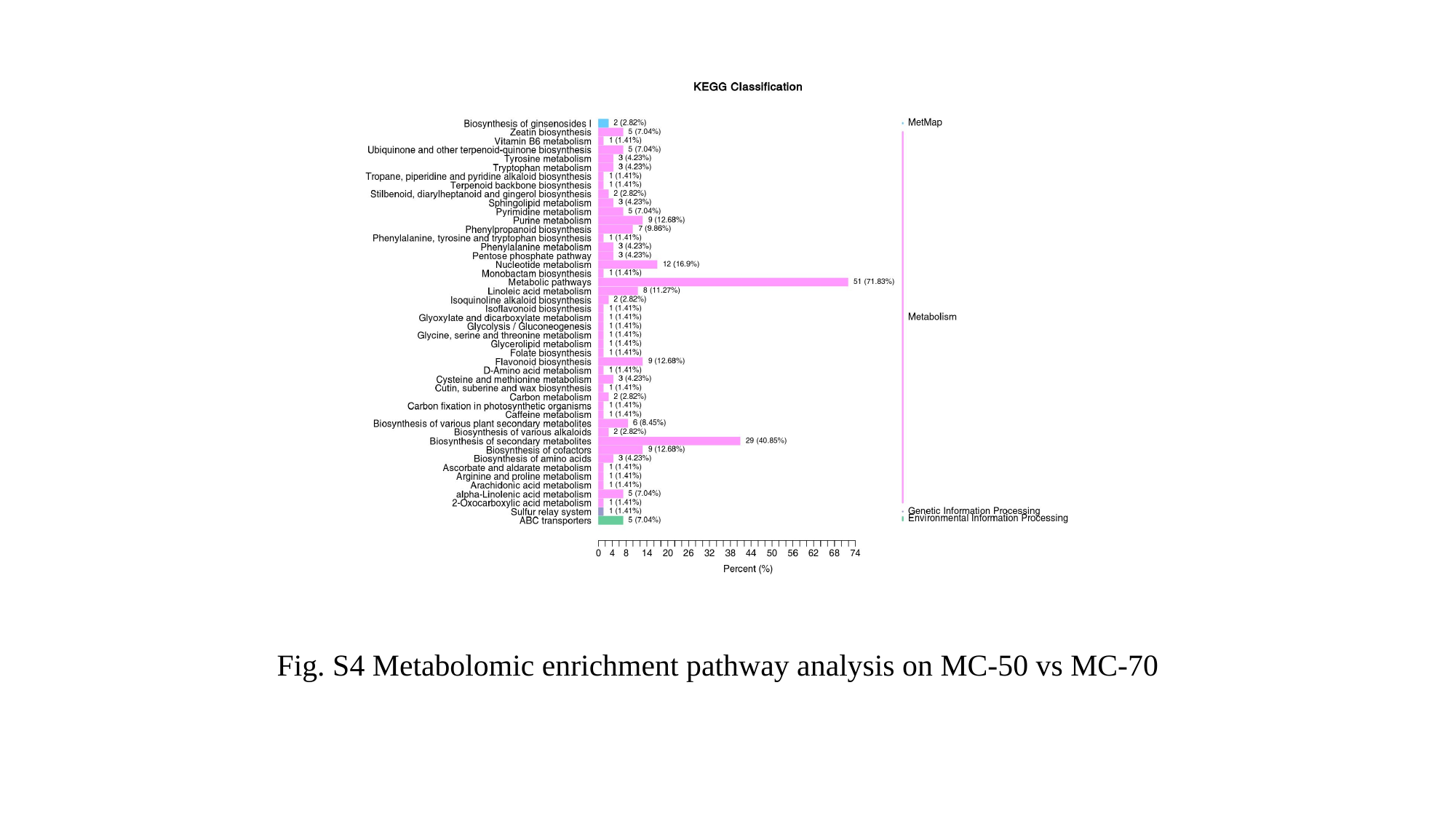

Fig. S4 Metabolomic enrichment pathway analysis on MC-50 vs MC-70

## Slide 5
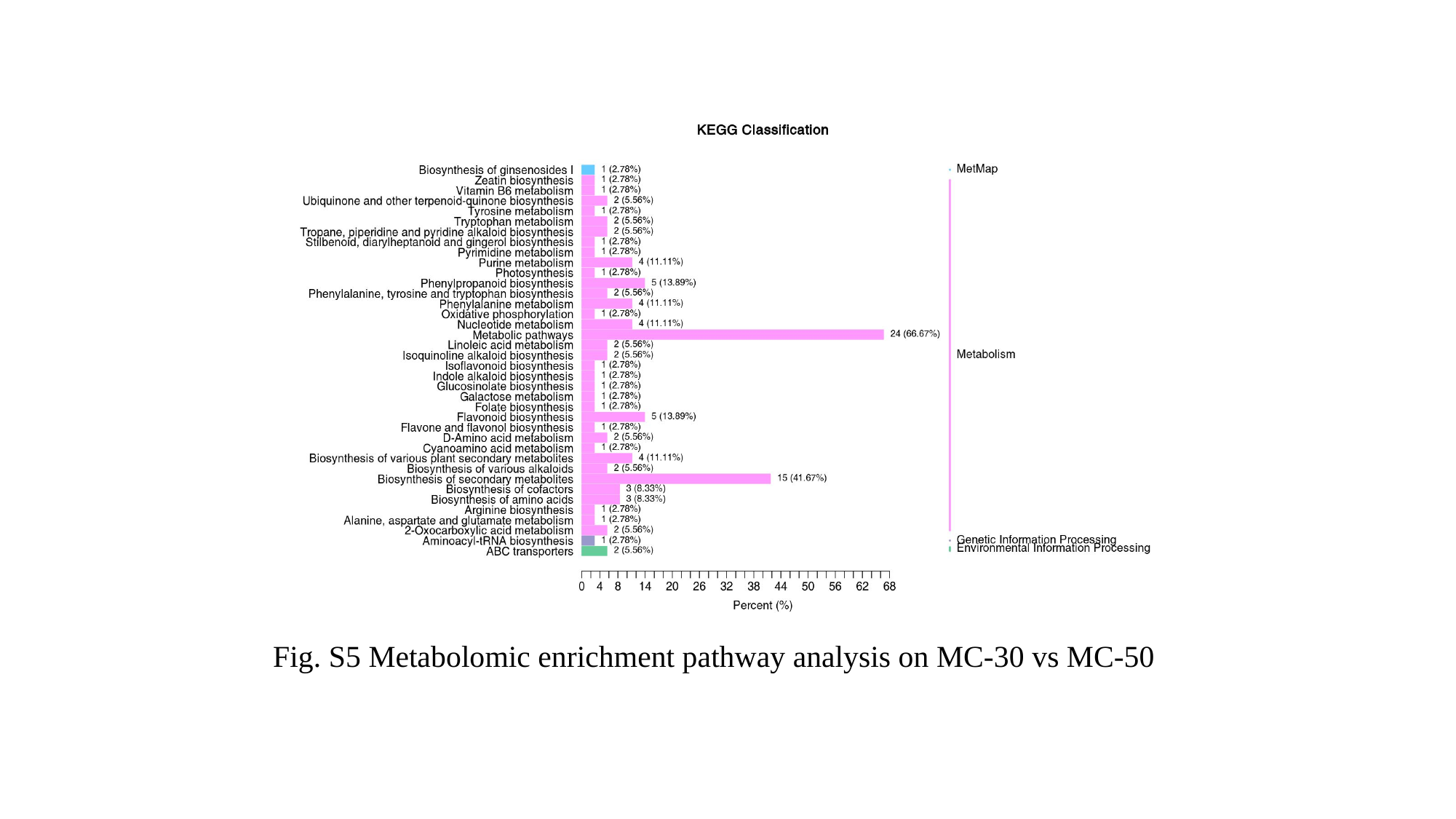

Fig. S5 Metabolomic enrichment pathway analysis on MC-30 vs MC-50

## Slide 6
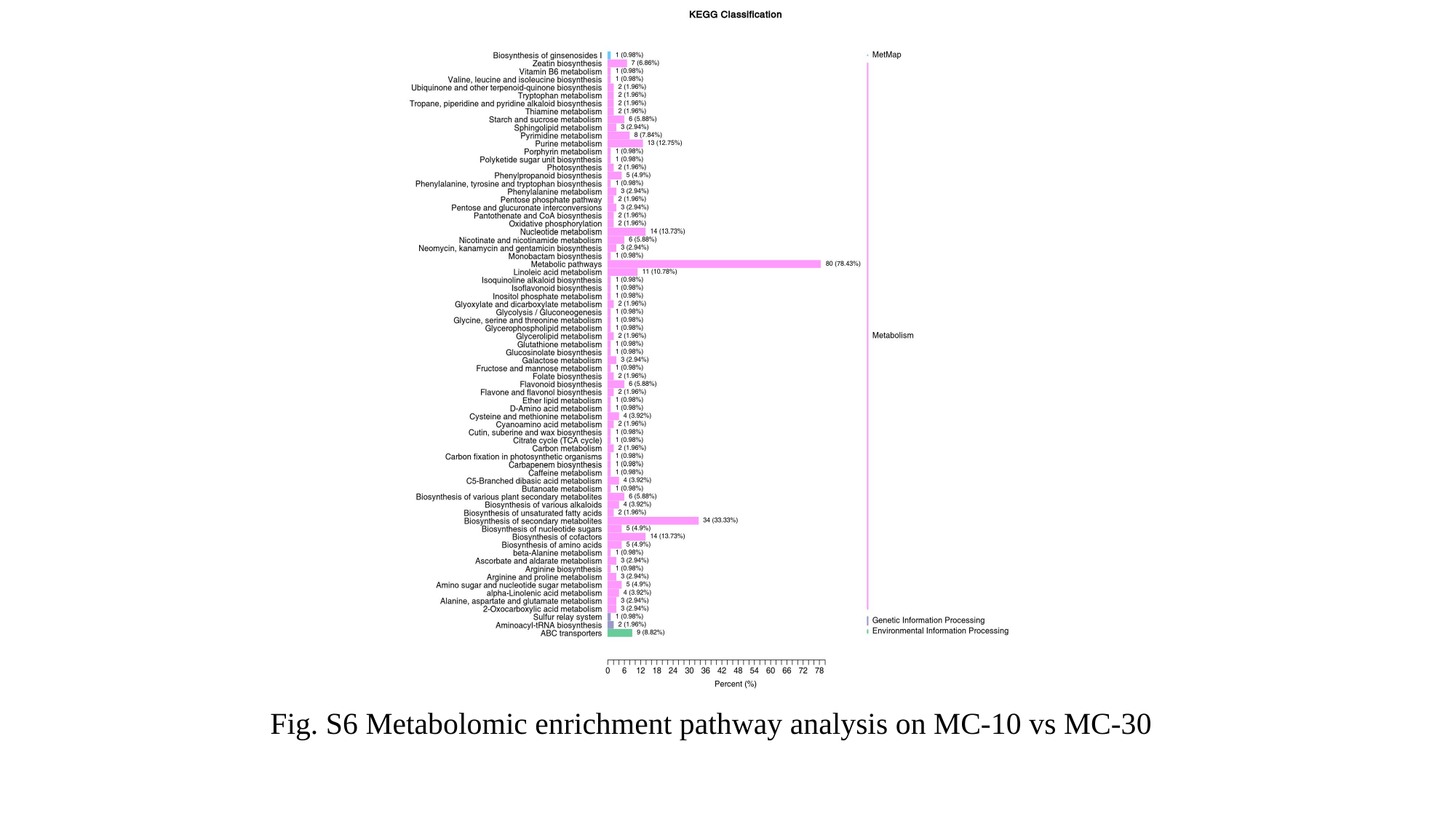

Fig. S6 Metabolomic enrichment pathway analysis on MC-10 vs MC-30
